# Homology search confirms widespread presence of BBSome proteins in Hexapoda with implications for potential non-ciliary BBS protein functions in honey bees

**DOI:** 10.1038/s41598-025-19137-w

**Published:** 2025-10-02

**Authors:** Alexander Ewerling-Haehnel, Ina Köhler, Isa Graebling, Anna Wierczeiko, Elisa Kotzurek, Susanne Gerber, Susanne Foitzik, Thomas J. Colgan, Helen L. May-Simera

**Affiliations:** 1 Institute of Molecular Physiology, Johannes Gutenberg University, Mainz, Germany; 2 Institute of Organismic and Molecular Evolution, Johannes Gutenberg University, Mainz, Germany; 3 Institute of Human Genetics, University Medical Center, Mainz, Germany; 4 Justus-Liebig-University, Giessen, Germany

**Keywords:** Cell biology, Computational biology and bioinformatics, Developmental biology, Evolution, Molecular biology, Zoology

## Abstract

Cilia were one of the characteristic traits of the last eukaryotic common ancestor and are highly conserved among eukaryotes. Their proteomic makeup is remarkably similar throughout all eukaryotic lineages. Recently, several ciliary transport proteins, namely the Bardet-Biedl Syndrome (BBS) proteins, were shown to traverse the nuclear envelope, and to modulate gene expression. Insects have been critically understudied in cilia biology since they only exhibit cilia on a subset of cells. We present evidence that the BBSome is largely conserved in multiple insect lineages. To examine BBS protein expression within insects, we profiled tissues, castes, and sexes of the honeybee *Apis mellifera*, a species where the genome encodes for multiple behavioural and morphological phenotypes. We find variation in expression profiles of putative BBSome-associated genes across different tissues, including those lacking cilia, indicating possible non-ciliary functions. We also demonstrate that expression of individual BBS proteins varies significantly between queens’ and males’ tissues, especially in neuronal tissue. Particularly high overexpression of BBS4 in glandular tissue indicates a cilia-independent role. Our findings provide evolutionary insight into the conservation of BBSome components across insects, suggesting potential additional roles for cilia proteins in non-ciliated tissues, providing candidate genes from diverse insect orders for future experimental work.

## Introduction

**Fig. 1:**
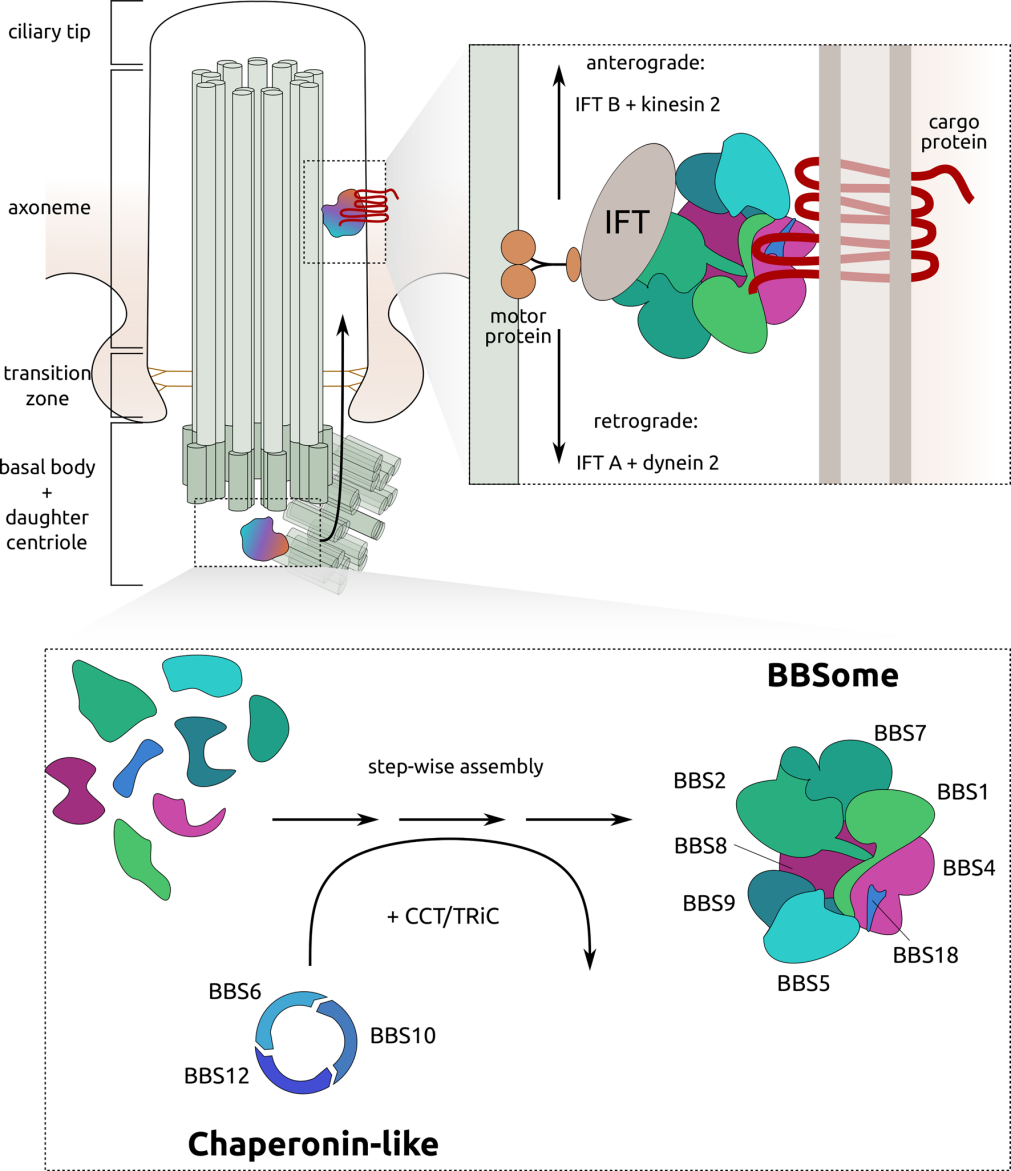
Ciliary functions are conserved in eukaryotes. (**A**) BBS proteins can be found at the ciliary basal body where the BBSome is assembled with chaperonin-like BBS proteins in concert with CCT/TRiC (chaperonin-containing t-complex protein 1/T-complex protein Ring complex) proteins. Ciliary cargo proteins are then transported along the axoneme with the help of IFT particles and motor proteins (adapted from^18,45,82^). (**B**) Although highly specialised, cilia can be found in insect neurons and sperm flagella. For orthologue search we assembled proteomes from several insect clades, and human and mouse as reference/outgroup.

Cilia are tiny hair-like microtubule-based organelles extending from the surface of most eukaryotic cells. These ancient organelles have a conserved structure, function, and proteome across eukaryotes^1,2^. They can be structurally divided into the basal body with a mother and daughter centriole which reside in the cytoplasm, the transition zone, the membrane-sheathed axoneme, and the ciliary tip^3^ (Fig. 1). While single-celled eukaryotes generally display motile cilia with dual functions of locomotion and sensory perception of the environment^2,4–6^, immotile (also termed “primary “) cilia are found as single copies per cell in metazoan cells^7–9^. Acting as a complex signalling centre, primary cilia are essential for several biological processes ranging from chemo- and mechanosensation to transduction of numerous signalling cascades, such as Notch, Hedgehog or WNT. Defects in cilia can cause several multisystemic diseases, referred to as ciliopathies, which show a wide variety of partly overlapping phenotypes^10–12^. One of these diseases, Bardet-Biedl Syndrome (BBS)^13,14^, represents a genetically heterogeneous inherited disease that is considered the archetypical ciliopathy since patients exhibit virtually all symptoms associated with ciliary dysfunction, albeit with different intensity. These include retinal degeneration, kidney malfunction, obesity, and mental retardation. BBS is caused by pathogenic mutations in genes encoding BBS proteins. Within the BBS proteins, two major subcomplexes are known in mammals: the BBSome and the chaperonin-like complex. The functionality of both is crucial to maintain ciliary trafficking and therefore, by extension, ciliary signalling^15–18^ (Fig. 1A).

Three main processes interact to make signalling pathways within cilia possible: trafficking of proteins to cilia from the cytoplasm, selective passage of cargoes at the base of the cilium (the so-called ‘transition zone’) into the ciliary lumen, and specific intraflagellar transport (IFT) along the ciliary axoneme^15,19,20^. The BBSome, a hetero-octameric complex consisting of the BBS proteins BBS1, BBS2, BBS4, BBS5, BBS7, BBS8, BBS9 and BBS18^17,18,21^, forms an adaptor that couples ciliary cargo proteins to motor proteins and IFT complexes. In addition to the transport of cargo to cilia, the BBSome is required to promote the retrieval and export of specific transmembrane proteins from the cilium^16,19,22–24^. The BBSome hence plays an important role as a key regulator of ciliary protein composition. A second characteristic group of BBS proteins, so-called chaperonin-like BBS proteins, is comprised of BBS6, BBS10 and BBS12, which show structural homology to the chaperonin-containing t-complex protein 1 (CCT) family of group II chaperonins^25^. It was previously demonstrated that these proteins form a hetero-oligomeric complex with other CCT proteins, and that this process plays a key role in the regulation of BBSome assembly^26,27^. In addition to the important role BBS and other ciliary proteins play in cilia, they have also been described to function in other cellular processes, such as cell cycle regulation^28–32^, and intracellular trafficking^33–35^, as well as regulation of gene expression by interacting with transcription factors, RNA polymerases, and DNA methylating proteins^36–40^ (for reviews, see^41–43^). Besides their ciliary functions, the biochemistry, and modes of action of BBS proteins outside of cilia are largely unknown.

One approach to further elucidate molecular mechanisms of ciliary proteins in non-ciliary functions is from an evolutionary perspective. Cilia are present in most eukaryotes; however, they were lost independently on multiple occasions in distantly related taxa^44^. Despite the absence of classical cilia, genes coding for putative BBS proteins were shown to still be present in some of the genomes of these organisms. A prime example is the parasite *Toxoplasma gondii*^45,46^ where cilia assembly occurs independently of the BBSome. The authors found that a single putative BBS5 gene homologue is still actively expressed in a non-flagellate state of the parasite’s life cycle, suggestive that this BBS-like protein does not perform cilia-associated functions and may have an alternative role. Consequently, these proteins might be essential beyond their ciliary functions. It is, therefore, of great interest to study the evolutionary conservation of putative BBS homologues and their expression in organisms that lack classical cilia. In this respect, insects are unique in the sense that most of their cells are not ciliated meaning that expression of BBS homologues may be associated with alternative non-canonical ciliary functions.

In arguably the most well studied insect, *Drosophila melanogaster,* the only ciliated cell types that have been identified are sensory neurons and motile cilia in sperm cells^47,48^. Neuronal cilia in *D. melanogaster* facilitate a variety of sensory perceptions: smell and taste are conveyed by chemosensory neurons in antennae, mouthparts, wings, and legs; hearing by mechanosensory neurons in the pedicle of the antennae; and proprioception by innervated bristles and campaniform sensilla all over the fly’s body^49–51^. Additionally, motile cilia can only be found in the sperm cells of male flies^52^. *D. melanogaster* has served as a model for developmental biology and genetics for over a century^53^ and has more recently come to the attention of cilia researchers due to a wide variety of genetic tools and behavioural assays available^54^. *Drosophila* assembles somatic neuronal cilia in a classical, IFT-dependent manner, whereas sperm cell ciliogenesis is IFT-independent^55,56^. The BBSome, which in other animals consists of eight components, lacks BBS2 and BBS7 in *D. melanogaster*^45,57,58^, and therefore has a likely different mode of action^59^. Due to the unusual composition of the BBSome in *Drosophila*, and potentially divergent evolution of other BBSome proteins^59^, we performed a comparative genomic analysis to identify putative BBS homologues and investigate evolutionary conservation across different insect orders, particularly since the restriction of cilia to certain tissue types presents an advantage in the search for non-ciliary functions in unciliated tissue. More specifically, our study focussed on the BBS proteins that form part of the BBSome, as well as the genes encoding the chaperonin-like BBS proteins. The BBSome is ancestrally conserved in ciliated species and was predicted to be present in the last eukaryotic common ancestor (LECA) while the chaperonin-like BBS proteins are restricted to the unikont lineage (which includes animals and fungi) and the basal eukaryote *Malawimonas jakobiformis*^45^ but are mostly found in metazoa.

At present, our understanding of evolutionary conservation and expression of BBS homologues in insects is largely restricted to *D. melanogaster* and other flies. To address this gap, we examined the conservation of putative BBS proteins across insects using a homology-based analysis incorporating members from diverse insect orders. As a complementary approach to understand expression patterns in putative BBS insect homologues, we examined gene expression in the western honeybee, *Apis mellifera*, which has a number of benefits for this purpose: 1) their genome was one of the earliest insect genomes to be sequenced and assembled^60^, with the species now having a wide range of transcriptomic and proteomic resources available^61,62^, making them an excellent non-model organism for explorative studies in cilia biology; 2) they are part of the social Hymenoptera, whereby caste differentiation has evolved within the female sex with differences in the expression of morphologically, behaviourally, and physiologically distinct phenotypes regulated by gene expression^63^; and 3) from an ecological and commercial pollination perspective, they represent one of the most important pollinators, essential for the maintenance of agricultural yields and wildflower diversity^64^ meaning understanding fundamental aspects of their biology is warranted. With respect to BBS proteins, a previous study has linked certain social behaviours to single nucleotide polymorphisms in a BBS1-like protein in wild populations of Cape honeybees^65^ demonstrating the species possesses certain homologues yet a detailed study of the conservation of putative BBS components, as their expression, in honeybees is currently missing.

Using publicly available datasets, we find evidence of the evolutionary conservation for the majority of the BBS components in different insect orders. Furthermore, to understand the expression profiles of such homologues, we investigated sex- and tissue-specific expression differences in honeybees allowing to pinpoint tissues where differential expression of BBS genes is most evident. Differential expression across different tissues may reflect localised specialisation of certain BBS components, which may point either diverged or additional functions beyond the traditionally viewed role in the BBSome.

## Results and discussion

### Homology-based search reveals conservation of BBSome proteins across insects

**Fig. 2:**
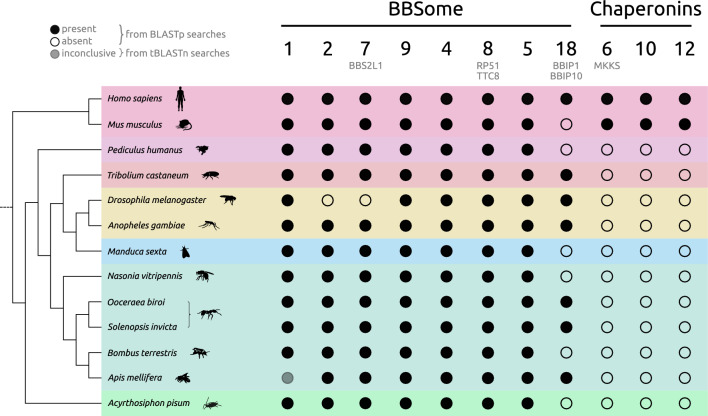
Evolutionary conservation of core BBSome genes across insects. Proteome-based searches uncovered putative insect homologues to most human BBSome proteins. The underlying gene model for BBS1 in the current *A. mellifera* genome assembly is likely a fusion of two neighbouring genes and thus does not produce orthologues in either BLASTp or OrthoFinder-based analyses, and is, therefore, greyed out and not considered in further analyses. Chaperonin-like proteins BBS6, BBS10, and BBS12 were, however, found restricted to humans based on our analysis.

BBS proteins are highly conserved across eukaryotes. In most homology-based searches, *D. melanogaster* has served as a representative of the insect lineage, along with a few others. In *D. melanogaster*, cilia and flagella are only found on sperm cells and neurons^48^. They still possess a functioning, relatively intact BBSome, albeit in a reduced form, with BBS2 and BBS7 missing from the otherwise complete complex found in other metazoa^45,57,58^. As the reliance of studies on single species or lineages may lead to biases through extrapolation, we, therefore, investigated if a partial reduction (or even complete loss) is common across other insect taxa, or if the loss of single components identified in *D. melanogaster* is the onset of a taxonomically-restricted gradual loss of cilia or at least components with putative functional roles in cilia. Therefore, the first aim in this study was to investigate the occurrence and distribution of BBS proteins across different insect lineages, including representative members from some of the most species rich orders. Generally, over large evolutionary distances, gene-based orthology searches are only a weak indicator of presence or absence of a given gene, as codon bias might heavily skew the analysis, whilst proteins are highly conserved on an amino acid level, but not necessarily on the nucleotide level. For protein-based homology searches, we, therefore, compiled a broad spectrum of predicted proteomes of diverse insects (n = 11 species representative of six orders; Fig. 2).

To understand evolutionary conservation of BBS proteins in insects, we performed protein-based homology searches between characterised BBSome and chaperonin-like BBS proteins in humans and a diverse (and explicitly not exhaustive) set of insect taxa, from Acercaria to Holometabola. For this approach, we obtained predicted proteomes from NCBI RefSeq and, for each species, conducted an all-versus-all BLASTp homology search. We found that homologues of genes coding for the BBS proteins that build the BBSome in humans (BBS1, BBS2, BBS7, BBS9, BBS4, BBS8, BBS5, BBS18) are present in almost all examined insect species (Fig. 2, Supp. Table S1). In contrast, none of the queried insect proteomes contained homologues for chaperonin-like BBS proteins BBS6, BBS10, or BBS12, on the basis of a reciprocal BLASTp. This lack of homologue detection may be due to loss of genes in insects, gain of genes in mammals or divergence of homologues beyond the point of detection. Given the early rise of chaperonin-like BBS proteins in Opimoda^45^, it is likely that these genes were lost early before the radiation of the insect families. As cilia are built in highly specialised cell types in insects, there may not be a need for specialised CCT-derived BBS proteins in these cells to aid the folding of the BBSome. Similar patterns were also identified using a complementary homology-based approach using OrthoFinder^66^, which constructs orthogroups consisting of putative homologues descending from a single gene in the last common ancestor of all examined species. Orthofinder also implements protein-vs-protein searches using DIAMOND^67^ (Supp. Figure 1A), and performs a normalisation step to remove potential introduced biases due to differences in protein length^66^. Phylogenetic reconstructions using OrthoFinder support a high degree of conservation within one insect lineage (e.g., within Hymenoptera), but also individual lineages that have highly divergent protein architectures (e.g. Diptera and Lepidoptera) (Supp. Figure 1B).

Overall, the high degree of conservation of the BBSome as a complex is reflected in most examined insect lineages, with *D. melanogaster* being a notable exception. BBS1-like proteins BBS2 and BBS7 were found to be missing in *D. melanogaster*, supporting previous studies that used reciprocal best BLAST (RBB) and Hidden Markov Model (HMM) searches^45,57^. Our analysis supports these losses in *D. melanogaster*. Interestingly, the only BBSome protein missing from *A. mellifera* in our protein-based search was BBS1. However, using tBLASTn, we found a partial match to a protein-coding gene on chromosome 2 (LOC410891). The resulting protein coded for a predicted functional domain associated with BBS proteins (IPR032728) yet manual inspection of the underlying gene model and its associated predicted protein revealed issues with the gene model. The gene model coded for a putative BBS1 but also coded for a protein ‘dispatched’ homologue, had more than twice as many exons (15 exons compared to seven) and as well as more than double the amino acid length (1612 aa vs. 583 aa) of homologues in closely related species, such as the bumblebee, *Bombus terrestris*.

This variation suggests that the gene model is a fusion of two neighbouring genes. Therefore, we tentatively conclude that a BBS1 homologue is present in the honeybee genome assembly but that the current gene model itself is incorrect. Other studies^65^ have identified a BBS1-like protein (LOC102655146) in Cape honeybees under selective pressure. Earlier homology searches also readily identified a BBS1 homologue in honeybees^57^. However, this gene appears to be missing in the latest annotations meaning either the gene model was misassembled, fragmented or a homologue does not exist in honeybees. The fact that we did not misidentify BBS2 and BBS7 in *D. melanogaster* does on the other hand speak for the sensibility of the approach. BBS1, BBS2, BBS7, and BBS9 all have similar protein architecture (N-terminal β-propeller, followed by an ɑ-helical linker and ɣ-adaptin-ear domain); BBS1 differs in that it is missing the platform- and C-terminal ɑ-helix domains present in BBS2, BBS7 and BBS9. The ultrastructure of the mammalian BBSome was recently established^18^, which suggests that BBS1 mediates the interaction between ARL6 and the BBsome needed for membrane attachment. Interaction is facilitated by the ɣ-adaptin-ear domain of BBS1, but that domain is also present in the other ‘BBS1-like’ proteins, so BBS1 itself may be substitutable in the BBSome of some species. However, this theory needs evidence on an experimental level and should be treated with caution.

### Single BBS proteins are differentially conserved across insect taxa

**Fig. 3:**
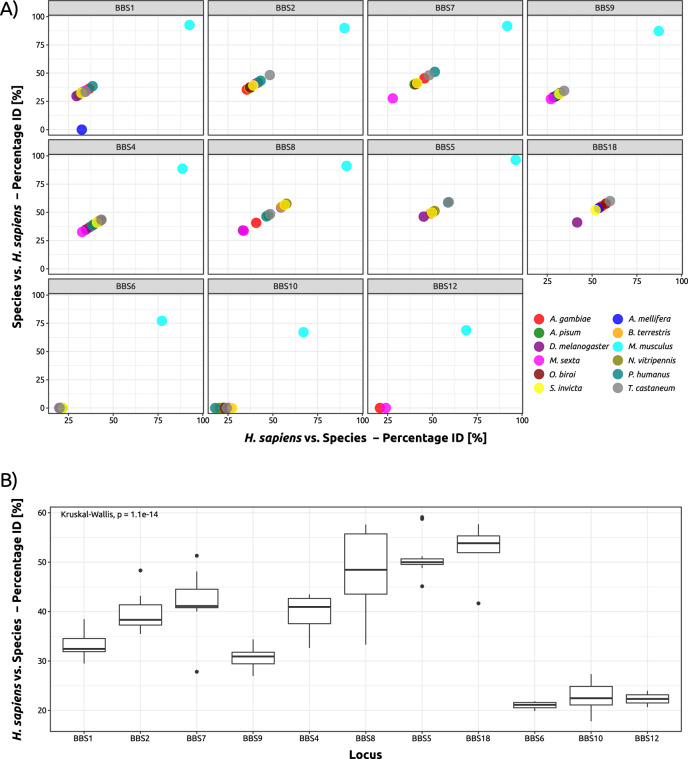
Variation in sequence similarity across insect BBS proteins. (**A**) Scatterplots for each identified BBS protein homologue across insect proteomes displaying percentage identity at the amino acid level between human BBS proteins (species 1) and putative homologues in insects (n = 11) and mouse (species 2). Each species is represented by an individual colour. (**B**) Boxplot displaying the distribution of percentage identify at the amino acid level for each BBS protein across all insect species examined (n = 11 species).

Overall, we found significant variation in terms of percentage identity shared between specific human BBS proteins and their insect homologues (min: 27.0% (BBS9); max: 60.0% (BBS18); Fig. 3A,B), which may reflect differences in terms of evolutionary conservation. While there is no single group of insects standing out in terms of conservation, there is a trend for individual BBS proteins generally being more conserved than others. The lowest sequence similarity among insect proteins to human BBS proteins was identified in BBS1 (29.5%), BBS7 (27.8%) and BBS9 (27.0%) homologues and highest in BBS5 (59.1%), BBS8 (57.6%) and BBS18 (60.0%) (Fig. 3A). There are seemingly some underlying trends in terms of sequence conservation (Fig. 3B): BBS proteins of the BBS1-like family (BBS1, BBS2, BBS7 and BBS9) have a comparably low sequence identity compared to human (medians between 30 and 45%), with BBS9 being the least conserved of the BBSome components on sequence level. The tetratricopeptide domain (TPR) proteins BBS4 and BBS8 ranked slightly higher (between 40 and 50%). BBS5 as a pleckstrin-homology (PH) domain protein and BBS18 as a small α-helical linker had the highest degree of conservation (both above 50%). While some homologues for chaperonin-like BBS proteins BBS6, BBS10, and BBS12 could be identified with low percent ID when seeding with human sequences (Fig. 3A), the reciprocal BLAST search only showed low similarity hits, deeming the homologues to be likely false-positives or spurious matches. Functional domain analysis of predicted proteins from putative insect homologues identified the presence of conserved BBS-associated domains (Supp. Table S1), further supporting predicted roles in the BBSome. Given the high degree of conservation of BBSome proteins, it is likely that the last common ancestor of insects also possessed a functional, complete BBSome, yet further examination using a larger, taxonomically diverse set of insects is required to validate this prediction. Low sequence similarities could reflect reduced constraints on functionality derived from specific amino acids, leaving room for evolutionary divergence and acquisition of novel functions. However, non-conserved structures may be amenable to loss, as could be the case for BBS1 in honeybees. The strikingly high sequence conservation of BBS5 on the other hand hints at potentially conserved functions within the BBSome. BBS8 has the most variation in sequence similarity of all BBSome proteins across insects (Fig. 3B). Assuming that the BBSome is in principle assembled in a stepwise manner comparable to the human BBSome complex^18^, it is surprising to find one of the ‘backbone’ BBSome components with such a high degree of sequence divergence. In turn, the altered structure of BBS8 in many insects could have influenced the evolution of other BBSome proteins, leading to novel architectures that differ from the human sequences to compensate the changes and maintain functionality. This in turn could have led to the acquisition of novel functions of individual BBS proteins outside the BBSome, at least for some of its components. Without experimental evidence of interacting domains of BBSome subunits, this claim is, however, only a potential explanation. Unlike for the human BBSome complex, the insect equivalent has not been confirmed biochemically and has merely been suggested due to the presence of homologues to human BBSome components; interactomic data is to this point still missing for its constituents (according to EMBL-EBI’s Complex Portal; accession number CPX-2747), complicating the prediction of functional homology for single proteins or even domains. Thereby, the influence of amino acid or domain changes on interactions between subunits or even co-evolution remains elusive.

However, some additional functions of BBS proteins are already known from mammalian models: In addition to its role in ciliary trafficking, BBS8 has been shown to play a crucial role in centrosome stability and cell division^68–70^. Putative non-ciliary functions could also be tissue-specific or depend on the developmental stage of the organism, independent of tissue. When searching for non-ciliary functions of BBS proteins, tissue-dependent expression likely plays a pivotal role in detectability. Previous studies showed a high probability for BBS1, BBS8, and BBS9 to be localised to the nucleus at some point and also showed a nuclear localisation for the human orthologues^36,45^, hinting at possible non-ciliary functions. This merits further investigation into where these proteins might be expressed, especially in respect to tissue- and sex-dependent differences.

### BBS expression is different in tissues of queens, workers and males

**Fig. 4:**
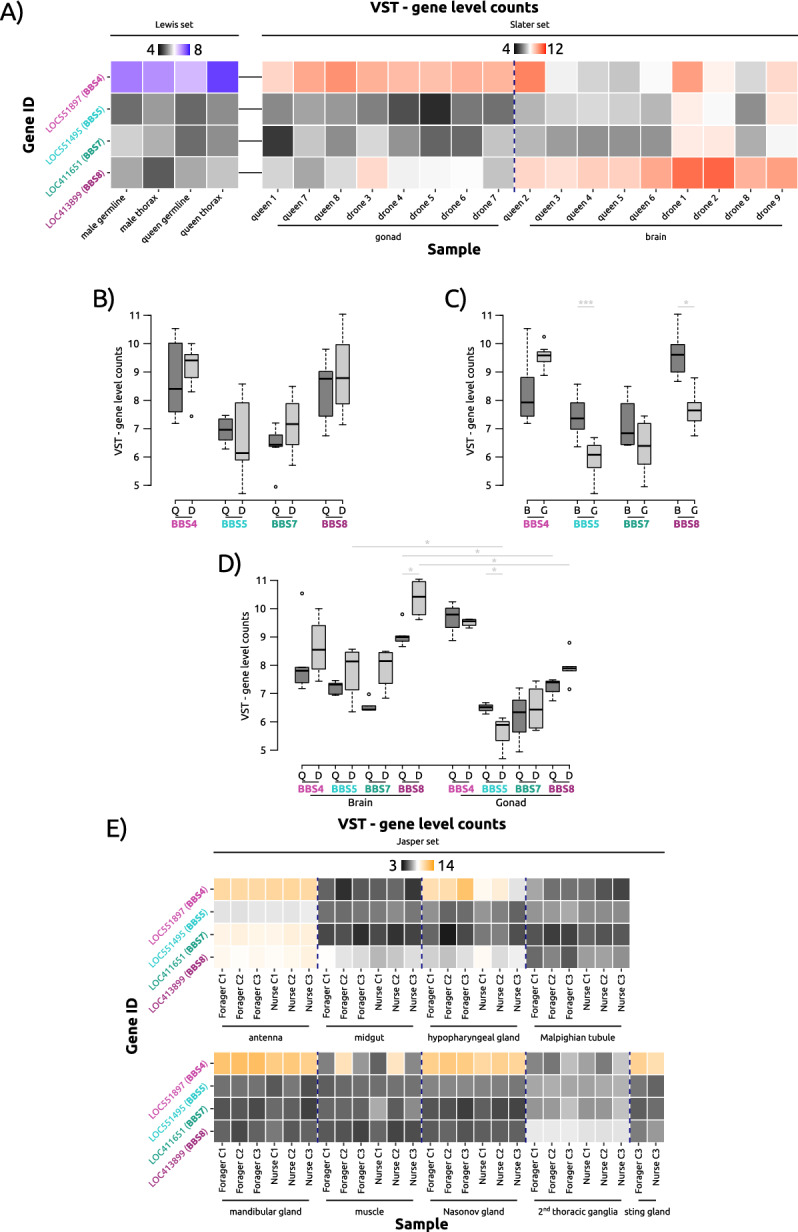
Expression differences of expressed honeybee BBS homologues from queens and drones, and across different tissues. (**A**) Variance-stabilising transformed (VST) gene level counts of BBS proteins in somatic and germline tissues of honeybee queens and males. Blue VST counts correspond to Lewis dataset^71^, red VST counts to Slater dataset^72^. (**B**) Expression profiles of BBS proteins in the brains and gonads of honeybee queens and drones (derived from Slater dataset^72^) (Welch’s test; n_queen_ = 8; n_drone_ = 9). (**C**) Expression profiles of BBS proteins across different tissues (derived from Slater dataset^72^) (Dunn’s test; n_brain_ = 9; n_gonad_ = 8; *: p <  = 0.05; ***: p <  = 0.001). (**D**) Expression profiles of BBS proteins across tissues of different sexes (derived from Slater dataset^72^) (Wilcoxon test; n_queen, gonad_ = 3, n_queen, brain_ = 5, n_drone, gonad_ = 5, n_drone, brain_ = 4; *: p <  = 0.05). (**E**) VST gene level counts of BBS proteins in somatic tissues of honeybee foragers and nurse (derived from Jasper dataset^79^). Q: queen, D: drone; B: brain, G: gonad.

As BBS expression is associated with neuronal tissues, we examined differential gene expression of putative BBS homologues across tissues and sexes of the honeybee, *A. mellifera*, using RNASeq data (Supp. Table S1). Given that honeybee queens and males (referred to as drones) differ extensively in terms of physiology, morphology, and behaviour, yet share the same genome, such observed phenotypic differences are largely regulated via differential gene expression. Using datasets consisting of RNASeq data of somatic and germline tissues from honeybee queens and males^71,72^, we identified the expression of BBS4, BBS5, BBS7, and BBS8 homologues in *A. mellifera*, with BBS4 being the most highly expressed overall (Fig. 4A). While in terms of overall expression, there were no significant differences between the sexes (Welch’s test; n_queen_ = 8; n_drone_ = 9; *p* > 0.05; Fig. 4B), we found significantly higher expression of both BBS5 and BBS8 in the brain compared to the gonads (Dunn’s test; n_brain_ = 9; n_gonad_ = 8; *p*_*BBS5, brain-gonad*_ = 0.0001, *p*_*BBS8, brain-gonad*_ = 0.0451; Fig. 4C). This is reminiscent of a tissue-specific gene expression pattern in mice, where even in ciliated tissues, BBSome components are not stoichiometrically expressed at the RNA level^73^. As there is no evidence on an experimental level that brain cells are ciliated in insects like honeybees or *Drosophila*^74^, and we cannot be certain of the purity of tissue preparation (i.e., to exclude ciliated tissue like spermatocysts or sperm cells), we can only speculate that the higher-than-average expression of BBS genes is due to mixed samples containing ciliated cells (like sperm cells and sensory neurons), or indeed a putative extraciliary function in these tissues. Yet future experimental validation is required to determine such a function. We then further compared the BBS RNA expression in tissues of queens and drones (both within the same sex and through comparison with the other sex; Fig. 4D) finding that BBS5 and BBS8 are significantly higher expressed in drone brains compared to drone gonads (Wilcoxon test; n_drone, gonad_ = 5, n_drone, brain_ = 4 Benjamini-Hochberg (BH) adjusted* p*_*BBS5, drone, brain-gonad*_ = *p*_*BBS8, drone, brain-gonad*_ = 0.0317). This pattern is the same for BBS8 expressed in queen brains versus queen gonads (Wilcoxon test; n_queen, gonad_ = 3, n_queen, brain_ = 5 BH-adjusted *p*_*BBS8, queen, brain-gonad*_ = 0.0357). When comparing expression in the brain, BBS8 is significantly lower expressed in queens compared to drones (Wilcoxon test; n_queen, brain_ = 5, n_drone, brain_ = 4 BH-adjusted *p*_*BBS8, brain, queen-drone*_ = 0.0357), and BBS5 is significantly higher expressed in queen gonads compared to drone gonads (Wilcoxon test; n_queen, gonad_ = 3, n_drone, gonad_ = 5; BH-adjusted *p*_*BBS8, gonad, queen-drone*_ = 0.0357). Differential expression using DESeq2 also revealed significantly higher expression of BBS8 in the brain of queens (LRT: Benjamini-Hochberg (BH) adjusted *p* = 6.72e-05) with a borderline difference in expression in the gonads (BH-adjusted *p* = 0.07; Supp. Table S1). Future work should include in situ staining of prepared brain tissue of both queens and drones to confirm that neuronal tissue is indeed non-ciliated in bees. Should this be the case, there is the possibility of non-ciliary functions of these proteins in brain tissue. In humans, BBS5 and BBS8 both localise to nuclei^45^ and both proteins have been shown to interact with E3-ubiquitin-protein ligase RING2 (RNF2)^36^, a protein of the polycomb group (PcG) repressor complex 1 (PRC1). This complex is mainly responsible for histone 2A (H2A) monoubiquitylation^75,76^, leading to repression of proteins crucial during development, such as those encoded for by homeobox genes^77,78^. Given the high degree of sequence conservation, this could also be the case in honeybees. BBS proteins thereby could potentially drive the development and sexual dimorphism between queens and drones in different ways.

An alternative, larger tissue RNA dataset^79^ consisting of different tissues from honeybee workers, and here, in particular, from nurses and foragers (Supp. Table S1), revealed a similar insight into expression profiles associated with neuronal tissues. As antennae are innervated by neurons bearing specialised primary cilia in *Drosophila*, we found a comparably higher expression level of BBSome components, as expected (Fig. 4E). Along with the eyes, the antennae are the most important sensory organs of the honeybee and have functions in chemosensory, tactile, temperature and acoustic perception. Type I and II cilia have been described for the Johnston organ of the honeybee antenna pedicel^80^, and cilia have also been found in the odour-detecting *sensilla placodea* at the tip of the antennae^81^. The higher transcript expression of BBS in antennae therefore may reflect a role in cilia function. However, BBS4, in particular, is strongly expressed especially in glandular tissues. The consistently high expression of BBS4 in all honeybee glands studied, including the hypopharyngeal and mandibular glands in the head of the worker bees, as well as the Nasonov and sting glands in their abdomen, suggests a specific function of this gene in glandular physiology. Indeed, BBS4 has also been implicated in intracellular microtubular transport, independent of the cilium^28^, a process that is indispensable for exocrine glands. However, to fully understand the possibly conserved role of BBS proteins in these tissues, further studies concerning their physiological function are required.

## Conclusion

The findings of this analysis predominantly validate earlier studies regarding the existence of BBS homologues in insects, such as *A. gambiae, A. mellifera,* and *D. melanogaster*^57,58^. While former studies focused on the evolution of molecular intraflagellar transport^58^ and the evolutionary history of the centriole from protein components^57^, our study investigated the evolutionary conservation of BBS protein homologues across a larger, yet non-exhaustive, set of insect species. Based on proteomic-informed homology searches, we were able to show that proteins forming the BBSome are potentially more conserved across insects than previously thought. Our study reveals that limiting homologue searches to few model organisms does not necessarily reflect the *status quo* across a larger set of species from the same family and might, therefore, skew conclusions that are drawn. In previous studies, homologues of all BBSome genes (with the exception of BBS2 and BBS7) were described at the genomic level in *A. mellifera* and *D. melanogaster*^45,57,58^. Through our analysis, we highlight that most genes are highly conserved, with the exception of BBS18, and appear as single-copies in many insect genomes. With increasing number of insect genomes, including improvements in gene annotation, future analyses incorporating a larger set of taxonomically diverse insects, as well as additional invertebrate outgroups, will provide a more comprehensive insight into the evolution of these important genes.

Our identification of conserved BBS protein homologues across diverse insect taxa points towards a theoretically fully functional BBSome in insects despite their deviation from the ‘classical’ function of (primary) cilia throughout different tissues seen in other animals. We also show that based on conserved homology and variation in gene expression across gene family members, honeybees provide an improved insect model to study BBS gene expression and potentially protein interactomics. Furthermore, using the honeybee as a study system, we find differences in gene activity of individual BBSome members across tissues, which may be due to possible cilia-independent functions. Our analysis provides candidate genes for future experimentation to determine the molecular functions that BBS protein homologues have in insects. Given the reduced occurrence of cilia in insect tissues, insects are a valuable model to further study the possible non-ciliary functions of BBS proteins, as their expression is not necessarily coupled to the presence of cilia according to the data presented here. The availability of high-quality transcriptomic data for different tissues and developmental stages of honeybees (and other insects) is an invaluable resource for future research into the unknown, cilia-independent functions of BBS proteins, and can be used to link genomic, proteomic, and behavioural phenotypes in an excellent, atypical model for cilia biology. Our analyses provide candidates for further examination through targeted experimental approaches and in situ expression analyses.

## Materials and methods

### Data procurement

To determine homology of BBS proteins across diverse insect orders, predicted RefSeq proteomes were downloaded from NCBI for the following species: Diptera, *Drosophila melanogaster*, *Anopheles gambiae*; Hymenoptera, *Apis mellifera*, *Bombus terrestris*, *Nasonia vitripennis*, *Solenopsis invicta*, *Ooceraea biroi*; Lepidoptera, *Manduca sexta*; Hemiptera, *Acyrthosiphon pisum*; Phthiraptera, *Pediculus humanus*; Coleoptera, *Tribolium castaneum*; and Mammalia, *Homo sapiens, Mus musculus*. While our choice of insects is non-exhaustive, we include representatives from some of the largest insect orders (e.g., Coleoptera, Hymenoptera, Lepidoptera, and Diptera). The data used for analyses of predicted transcripts and genome assemblies (Supp. Figure 2) was obtained from the NCBI nucleotide (nt) database. For each insect, the representative genome and predicted transcripts encoded by GenBank assemblies were used for this purpose. A list of the GenBank assembly accessions can be found in Table S1h.

### BLAST analysis and homologue search using OrthoFinder

A reciprocal BLASTp^83^ (e-value cut-off = 0.001) search was performed between *H. sapiens* and the insect species of interest. For each BBS gene and each targeted insect species, the result with the highest bit-score was chosen. These hits were then examined and extracted from the reciprocal BLASTp search for each putative insect BBS homologue that matched an individual characterised human BBS protein. As a complementary approach, for each species, the longest predicted peptide was extracted using the OrthoFinder script primary_transcript.py^66^. OrthoFinder was subsequently used (default settings) to perform pairwise alignments between each species allowing for the identification and generation of orthogroups, which are groups consisting of potential homologues across species of interest. Such orthogroups were parsed for the determination of insect homologues of described human BBS genes. Lastly, if an insect homologue was not identified by either reciprocal BLASTp searches or by OrthoFinder, which implements all-vs-all protein alignments using DIAMOND, we aligned the human BBS protein against the insect genome assembly using tBLASTn.

In terms of the BLASTn analysis^84^ a hit was defined by a detected significant similarity between the sequences compared with respect to the chosen parameter values. Hits can differ in aspects like position, length, e-value, and score. In this study the e-value was chosen as representation value to classify the significance and display hits in our BLASTn analysis of BBS gene expression. The BLASTn analyses were carried out via the NCBI BLAST online tool (general parameters: expect threshold = 0.05, word size = 11; scoring parameters: match/mismatch scores = 2,-3, gap costs = existence: 5 extension: 2).

### Differential gene expression analysis

For the purposes of examining gene expression of putative BBS homologues, we examined expression profiles in somatic and germline tissues of the Western honeybee, *A. mellifera*^79^, BioProject ID: PRJNA243651;^71^, BioProject ID: PRJNA386859;^72^, BioProject ID: PRJNA689223). We downloaded publicly available transcriptomic datasets from the NCBI Short Read Archive using the sra-toolkit. For each library, we extracted the data in FASTQ format and performed quality assessments using FastQC v.0.11.9 (https://www.bioinformatics.babraham.ac.uk/projects/fastqc/). Data were filtered using fastp v.0.23.2^85^ to remove low quality reads and trim adaptors. We then pseudoaligned each sample against an indexed predicted transcriptome using kallisto v.0.44.0^86^ providing a transcript-level quantification of expression. Using these estimates, we generated gene-level counts using tximport v1.26.1^87^ and for each dataset, generated variance-stabilised transformed data using DESeq2 v1.38.3^88^.

### Statistical testing

Datasets used for statistical testing can be found in Supp. Table S1. Tests were performed in R v.4.0.3^89^, with packages tidyverse^90^ (including dplyr^91^, ggplot2^92^ and stringr^93^), reshape2^94^, and FSA^95^. The accompanying Rmarkdown notebook is available on figshare (10.6084/m9.figshare.25144439). Datasets were tested for normality by Shapiro–Wilk testing (where normality was assumed if p > 0.05). Depending on normality and homoscedasticity, statistical tests were performed using Welch’s test (normally distributed, no assumption of homoscedasticity), Dunn’s test (no normal distribution, no assumption of homoscedasticity) or Wilcoxon rank-sum test (no normal distribution, used for selected multiple comparisons) (p > 0.05: not significant; 0.05 >  = p > 0.01: *; 0.01 >  = p > 0.001: **; 0.001 >  = p > 0.0001: ***). Multiple testing correction was done using the Benjamini–Hochberg procedure^96^. As a complement, we also implemented a DESeq2-based differential expression analysis to compare expression values using likelihood ratio tests with the results provided in Supp. Table S1.

### Additional software

Figures were prepared with ggpubr^97^ and Inkscape^98^.

### Further information

The accompanying GitHub repository will be available at date of publication.

## Supplementary Information

Supplementary Information 1.

Supplementary Information 2.

Supplementary Information 3.

Supplementary Information 4.

## Data Availability

All the genome assemblies and associated predicted proteomes used for the present analyses are publicly available from the National Center for Biotechnology Information (NCBI) GenBank database, with the full list of genome assemblies provided in the supplementary information (Supp. Table S1). The RNA-seq data of A. mellifera used in the present study are publicly available from the NCBI Sequence Read Archive (SRA) database (BioProject Accession: PRJNA689223; PRJNA243651; PRJNA386859).
