# Supplementary figures and images for: Homology search confirms widespread presence of BBSome proteins in Hexapoda with implications for potential non-ciliary BBS protein functions in honey bees
Source: Sci Rep. 2025 Oct 2;15:34312. doi: 10.1038/s41598-025-19137-w (PMC12491567; doi:10.1038/s41598-025-19137-w)

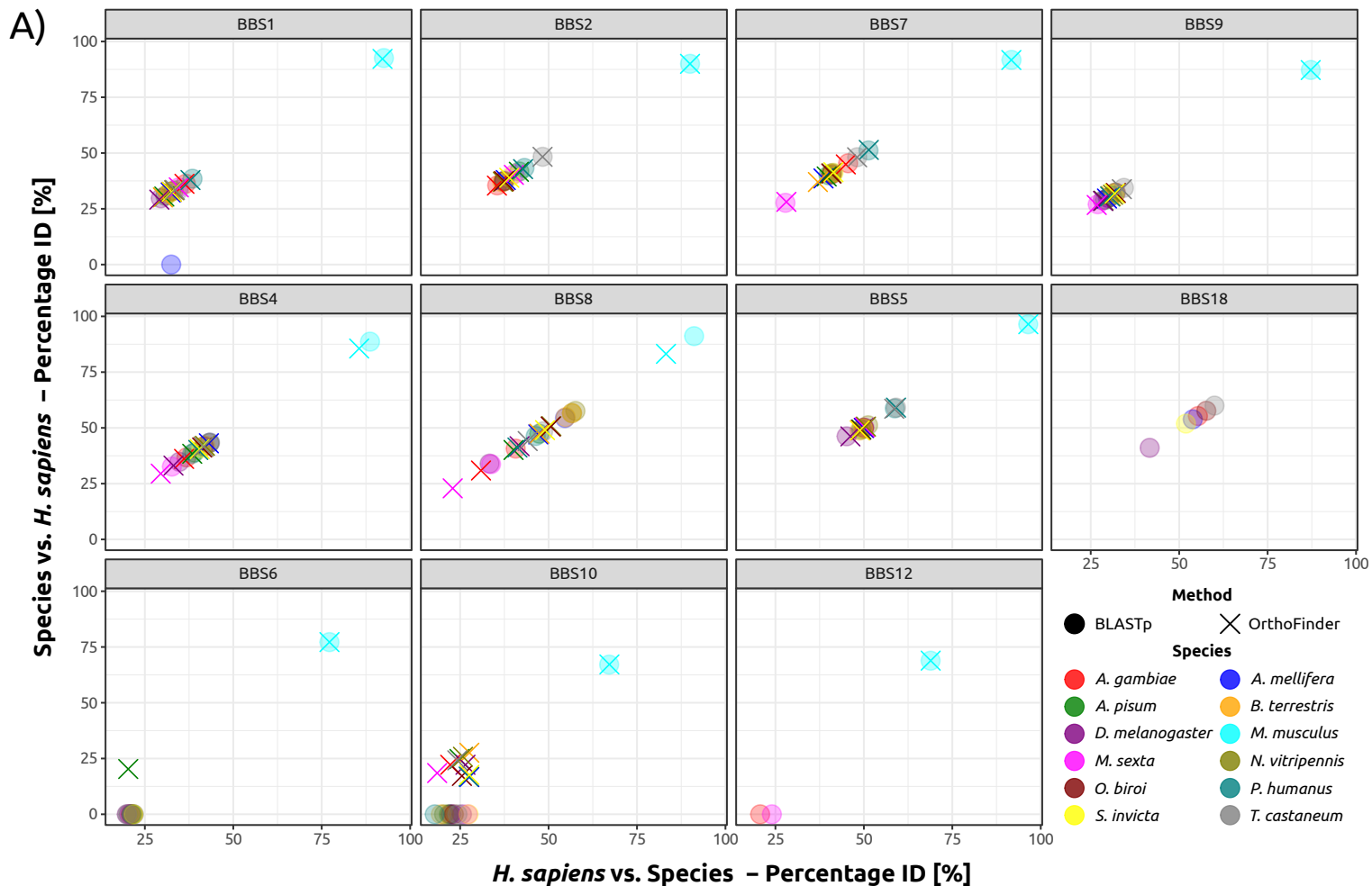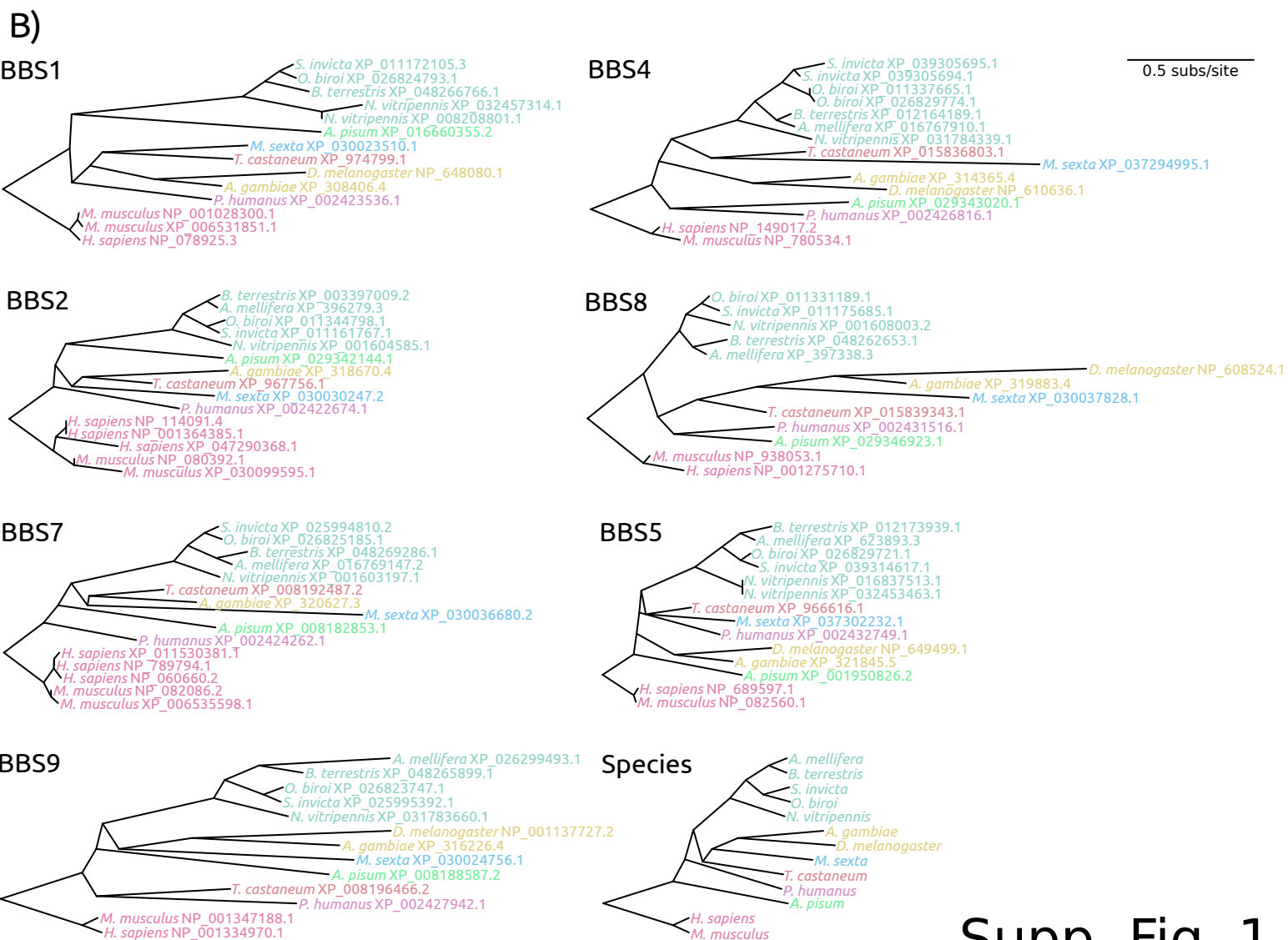

Supp. Fig. 1

Supplement: Supplementary file 1 — Supplementary Information 1. [file 41598_2025_19137_MOESM1_ESM.pdf]

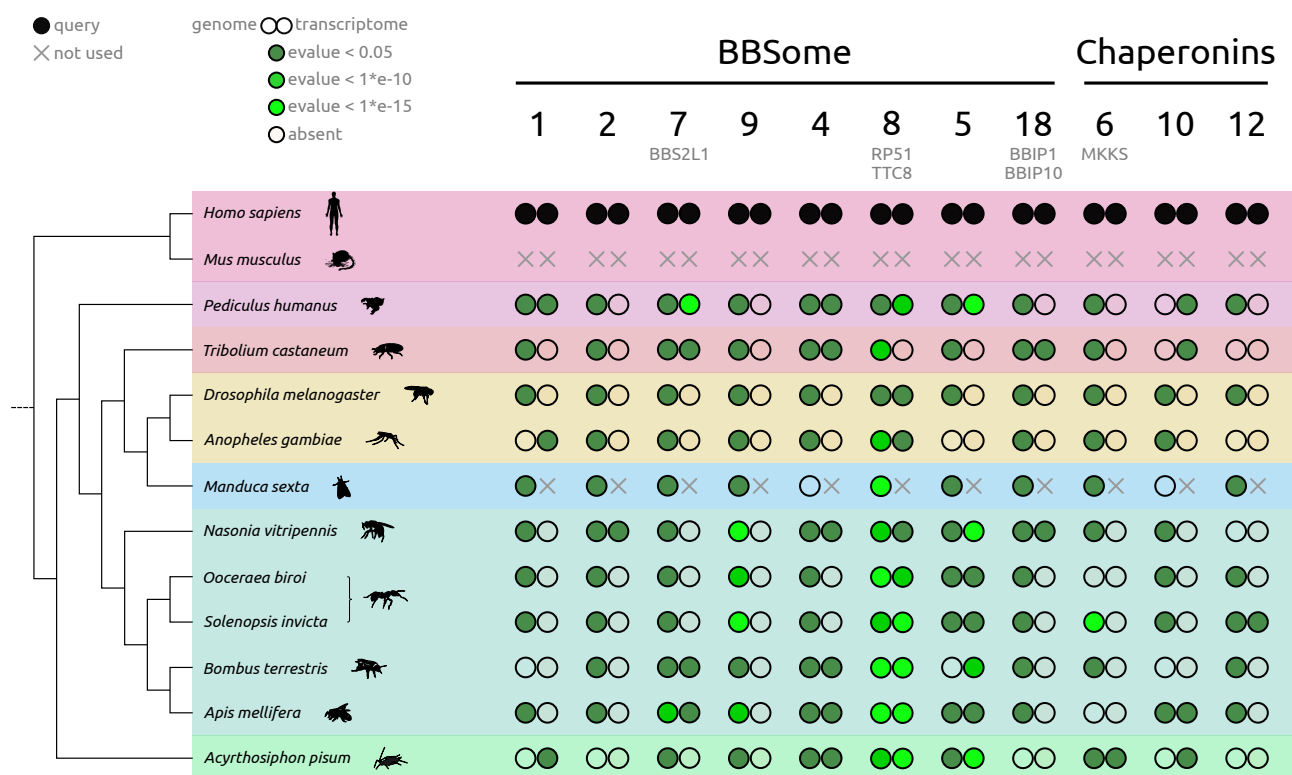

Supplement: Supplementary file 2 — Supplementary Information 2. [file 41598_2025_19137_MOESM2_ESM.pdf]
