# Supplementary material for: Homology search confirms widespread presence of BBSome proteins in Hexapoda with implications for potential non-ciliary BBS protein functions in honey bees
Source: Sci Rep. 2025 Oct 2;15:34312. doi: 10.1038/s41598-025-19137-w (PMC12491567; doi:10.1038/s41598-025-19137-w)
Supplement: Supplementary file 4 — Supplementary Information 4. [file 41598_2025_19137_MOESM4_ESM.docx]

SUPPLEMENTARY LEGENDS

Supp. Fig. 1: **Comparison of orthologue search strategies OrthoFinder and BLASTp.** A) Scatterplots for each identified BBS protein across insect predicted proteomes displaying percentage identity at the amino acid level between human BBS proteins (species 1) and putative homologues in insects (n = 11) and mouse (species 2). Each species is represented by an individual colour while the tool used for the basis of comparisons is indicated by shapes: Crosses, OrthoFinder; circles, BLASTp. B) Phylogenetic relationships between putative homologues identified via OrthoFinder. Leaf nodes are colour-coded to represent lineages as laid out in the ‘Species’ tree (and Fig. 2).

Supp. Fig. 2: **Comparison of genome and transcriptome searches using BLASTn.** Compared to proteomic searches (Fig. 2), genome searches are easily hitting false-positives and produce spurious results (hits for almost all chaperonin-like BBS proteins). Interestingly, transcriptomic analysis does in some cases indicate a transcribed gene that is not present in the genomic searches.

Supp. Table S1: Output files for homology-based BLAST searches, OrthoFinder-based analyses, as well as transcript abundances for honeybee tissues.
